# Investigating Physiology‐Behavior Associations for Youth During Parent–Child Conflict Discussions

**DOI:** 10.1002/dev.70149

**Published:** 2026-03-31

**Authors:** Salome Vanwoerden, Vera Vine, Amy L. Byrd, Kathryn B. Altman, J. Richard Jennings, Stephanie D. Stepp

**Affiliations:** ^1^ Department of Psychiatry University of Pittsburgh Pittsburgh Pennsylvania USA; ^2^ Department of Psychology Queens University Kingston Ontario Canada; ^3^ Department of Human Development and Quantitative Methodology, National Institute of Mental Health, Section on Development and Affective Neuroscience University of Maryland College Park Maryland USA

**Keywords:** observed behavior, parent–child conflict, respiratory sinus arrhythmia

## Abstract

Adolescents’ observed affective behavior is associated with patterns of concurrent physiological responding, which explain clinically relevant outcomes. Few studies have elucidated the physiology‐behavior association during the developmentally salient context of conflict with parents. The current study evaluated associations between respiratory sinus arrythmia (RSA) activity and observed youth affective behavior during parent–child conflict. We took a contextually informed approach to address limitations of past research with a clinical sample of parent–child dyads (*N* = 162, *M*_youthage_ = 12.03 (0.92) years; 46.9% female; 42% White and non‐Hispanic/Latinx; 40.7% Black; 16.7% multiracial). Dyads completed a conflict discussion during which youths’ affective behaviors were observationally coded. Additionally, we measured youths’ RSA and subjective affect as well as parents’ negative behavior. Youths’ angry/defiant behavior was predicted by faster RSA withdrawal, increases in subjective negative affect, and more negative parent behavior, whereas youths’ sad/distressed and positive engagement behaviors were predicted solely by subjective negative affect and negative parent behavior, respectively. Findings underscore the importance of taking context into account to understand the physiology‐behavior association among youth in developmentally salient interactions, which has implications for enhancing etiological models of psychopathology.

## 1 Introduction

Parent–child conflict is a central task of the transition to adolescence (Smetana and Rote 2019). With the drive for individuation and autonomy that youth experience during this period, parent–child conflict increases in frequency and intensity (Branje 2018). Although conflicts can be negatively charged, when youth effectively engage (i.e., cooperate and express positive affect) rather than avoid conflict, positive outcomes are observed (Ubinger et al. 2013). Alternatively, problematic behaviors, such as angry/defiant behavior during parent–child conflict, relate to the development of psychopathology among youth (Marceau et al. 2015). The current study explored the physiological underpinnings of observed youth behavior during parent–child conflict, specifically considering the influence of respiratory sinus arrhythmia (RSA). Although RSA activity has been tied to various psychopathological outcomes (Beauchaine et al. 2019), its role is context‐specific and must be understood in the developmentally salient context of parent–child conflict. This study aimed to unpack the physiology‐behavior association unique to parent–child conflict by considering the additional contextual factors of youths’ subjective experience and parents’ behavior. By understanding the physiological mechanisms underlying youths’ behavior during conflict, we can shed light on the role these mechanisms play in psychopathology development during adolescence.

### 1.1 Youth Affective Behavior and Its Physiological Correlates During Parent–Child Conflict

Understanding youth affective behavior during parent–child conflict is important, particularly during the transition to adolescence. In this period, youth experience intensifying emotionality (Bailen et al. 2019) that coincides with a move toward individuation from parents, a key developmental task (Cotterell 2013). Increasing conflict with parents represents a context for youth to assert themselves and renegotiate the relationship (Branje 2018). Youths’ observed affective behavior during conflict (i.e., anger, sadness, and positive engagement) functions as self‐expression and impacts successful navigation of essential developmental tasks (Moed et al. 2015; Van der Giessen et al. 2015). *Expressions of anger and defiance* by youth represent normative attempts to assert their needs (Sears et al. 2014). Similarly, youths’ expressions of *sadness or distress* are developmentally important as a means of self‐expression and communication of one's viewpoint (Rotenberg et al. 1988), which can be adaptive to de‐escalate conflict (Ferrar et al. 2020). However, excessive or persistent expressions of *anger* and/or *sadness* can escalate conflict (Ferrar et al. 2020) and are associated with psychopathology (Laursen and Collins 2009; Yap et al. 2011). It is beneficial to balance negative emotion expressions with *positive engagement* to maintain closeness and foster resolution (Laursen and Collins 2009). Indeed, expressions of positive engagement during interactions reflect collaboration to reach resolution (Missotten et al. 2018). Increased expressions of negative affect have the effect of reducing flexible expressions of affect, a component of effective emotion regulation and thus a transdiagnostic marker of psychopathology (Gonzalez‐Escamilla et al. 2022).

Few studies have characterized complex, clinically relevant behavior in adolescents in terms of concurrent physiological responding, or the more implicit phenotypes that may underlie or help explain it. Affective behaviors are partially guided by physiological activity (Nord and Garfinkel 2022). According to Polyvagal Theory (Porges 2007), parasympathetic vagal activity increases when the environment is perceived to be safe and functions to regulate cardiovascular activity in a way that promotes energy conservation and restoration. By contrast, vagal influence decreases in challenging or threatening contexts, supporting mobilization of resources and minimizing social engagement. RSA, an index of cardiac vagal influence on the heart, is estimated as the variability in time series of consecutive heartbeats synchronized with respiration (Quigley et al. 2024). RSA has been interpreted as reflecting an individual's physiological capacity to respond flexibly to environmental demands (Hastings et al. 2008; Muhtadie et al. 2015). During challenging parent–child interactions, RSA initially withdraws, possibly reflecting a narrowing of attention or readiness for challenge (Glackin et al. 2021). This normative pattern during a parent–child self‐disclosure task has been associated with desirable traits in unselected adolescents, including self‐reported prosocial behavior and affect regulation (Cui et al. 2015). Conversely, deviations from this pattern may be maladaptive; RSA activity during various tasks has been associated with a range of youth psychopathology across multiple studies (Beauchaine et al. 2019). However, these deviations are heterogenous and sometimes inconsistent (e.g., Calkins et al. 2007; Hinnant and El‐Sheikh 2009), with variability due to the types of stimulus conditions (with associations observed in negative emotion induction tasks) and observed across the internalizing and externalizing spectrum (Beauchaine 2015; Beauchaine et al. 2019; McLaughlin et al. 2014; Obradović 2012). Synthesizing these findings, a somewhat recent meta‐analysis observed greater RSA withdrawal to challenges among youth with externalizing problems with less consistent findings in regard to associations with internalizing problems (Beauchaine et al. 2019).

Delineating physiology‐behavior associations can help illuminate the self‐regulatory function of adolescents’ behaviors and the role of physiological self‐regulation in clinical outcomes. However, given the context‐sensitive nature of RSA activity, it is useful to evaluate the associations between changes in RSA and youths’ affective behavior in specific, clinically relevant populations and contexts. In an adult sample, RSA withdrawal predicted sociability during a positive social context and negative behavior in the context of rejection (Muhtadie et al. 2015). In youth, faster RSA withdrawal was associated with observed negative affect and behaviors during dyadic challenges (Crowell et al. 2014; Glackin et al. 2021). Conversely, RSA augmentation (increases) in response to dyadic challenge—the unexpected response in contexts requiring vigilance or effort (Porges 2007)—has been associated with social disengagement (Lunkenheimer et al. 2018; Thayer and Lane 2000). However, only a few studies have characterized change in RSA in relation to youths’ affective behavior during parent–child conflict. In a sample of clinically depressed adolescents, RSA withdrawal was associated with more aversive youth behavior (i.e., coercive or attacking utterances) (Crowell et al. 2014), whereas in a community sample, there was no association between youths’ observed negative affect and momentary RSA (McKillop and Connell 2018). Thus, more research is needed to understand the physiology‐behavior association during parent–child conflict, especially among dyads at higher psychopathology risk.

### 1.2 Additional Correlates of Youth Affective Behavior

Beyond physiological responding alone, other affect‐related variables could also be associated with youths’ affective behavior during parent–child conflict. Accounting for these other variables using a contextual, multimodal lens may bring additional specificity to our understanding of the role of physiological responding in youths’ conflict behavior. First, subjective affect provides important contextual information about parent–child conflict, representing one's perceptual experience of the interaction. As parent–child conflicts are highly unique within families, including a measure of youths’ subjective affective experience can help control for this variability. Surprisingly, only a single study measured youth RSA during parent–child conflict alongside subjective youth affect. While not analyzed together, they found that subjective experiences tracked with observed affective behavior, whereas youth RSA did not demonstrate unique associations with any type of behavior (Kaufman et al. 2020). However, no studies have examined whether physiological and subjective experiences exert unique effects on youth affective behavior during parent–child conflict.

Relatedly, and in line with theory, suggesting RSA is sensitive to safety signals in the environment, accounting for parent behavior during conflict may also add important contextual information when evaluating the links between RSA and youths’ affective behavior (Kaufman et al. 2020). Indeed, adolescents’ RSA during a conflict discussion was predicted by mothers’, not adolescents’, expressions of negative affect (McKillop and Connell 2018). Similarly, child‐initiated repair behaviors were unrelated to their own RSA after accounting for mothers’ behaviors in a preschool‐aged sample while completing a challenging puzzle (Lunkenheimer et al. 2019). Thus, it may be that RSA reflects reactivity to the social context, but not actual social engagement. However, additional research is needed that accounts for both subjective and contextual aspects of parent–child conflict to further elucidate the association between physiology and behavior.

### 1.3 Current Study

This study aimed to identify associations between RSA activity and youths’ observed affective behavior, specifically *anger/defiance*, *sadness/distress*, and *positive engagement* during parent–child conflict while controlling for youth subjective negative affect and observed negative parent behavior. Thus, we examined how different modalities (i.e., youth RSA, youth subjective affect, and observed parent behavior) functioned as parallel predictors for youth observed affective behavior. We tested this question in a clinical sample, given the implications of RSA reactivity as providing context for psychopathological outcomes in youth and to provide greater rates and variability of parent–child conflict. Transdiagnostically, RSA withdrawal has been linked to self‐control, and emotion and behavior dysregulation (Byrd et al. 2022; Graziano and Derefinko 2013) and associations between RSA reactivity and clinical outcomes are enhanced in clinical samples (Graziano and Derefinko 2013). However, the mechanism by which RSA reactivity predicts risk for psychopathology can be further clarified by understanding the RSA‐behavior association during parent–child conflict, which has known implications for child psychopathology and is a malleable intervention target (Orte et al. 2025). Further, studying the role of RSA reactivity during emotionally salient contexts in a clinical sample enhanced for emotion dysregulation allows for greater variability in ineffective conflict behaviors and can guide prevention and intervention efforts for youth at the highest risk for developing psychopathology. We expected to observe RSA withdrawal during conflict anticipation (Poole and Schmidt 2021; Renna et al. 2025) and conflict discussion (Crowell et al. 2014; Cui et al. 2015; Glackin et al. 2021), reflecting an adaptive response to a challenging social environment (Cui et al. 2015; Miller et al. 2013), which would predict both more *anger/defiance* and *positive engagement*. We also expected that greater youth subjective negative affect and observed negative parent behavior would predict more *anger/defiance* and *sadness/distress*, as well as less *positive engagement*. However, given that no previous studies have taken a multimodal approach when evaluating physiology‐behavior associations, we had no a priori hypotheses about the unique effects that may emerge once accounting for additional contextual variables.

## 2 Methods

### 2.1 Participants

Parent–child dyads (*N* = 162; *M*_youthage_ = 12.03, SD = 0.92 years; 46.9% female) were recruited from primary care and ambulatory psychiatric treatment clinics. To be eligible, youth had to be receiving psychological or psychiatric services in any settings. Additionally, youth were oversampled for emotional reactivity using the Affective Instability subscale of the Personality Assessment Inventory‐Adolescent version (Morey 2007). Table 1 lists the rates of diagnoses observed in the sample based on the Schedule for Affective Disorders and Schizophrenia for School‐Age Children (K‐SADS; Kaufman et al. 2020) and Childhood Interview for Borderline Personality Disorder (CI‐BPD; Zanarini 2003). Most caregivers were female (92.3%) and biological parents of the child (94%). All caregivers had legal and primary physical custody. Dyads were racially and socioeconomically diverse, with 42% of adolescents White and non‐Hispanic/Latinx; 40.7% Black, 3.7% Hispanic/Latinx, 0.6% American Indian or Alaskan Native, and 16.7% multiracial. Over 50% of the sample reported annual family incomes under $60,000, with 30.9% reporting less than $20,000, and about half of the sample reported receiving public assistance (e.g., Special Supplemental Nutrition Program for Women, Infants, and Children [WIC], food stamps, or welfare). Participants were recruited between 2015 and 2018.

**TABLE 1:** Prevalence rates of youth diagnosis.

Disorder	% with diagnosis
Attention deficit hyperactivity disorder	62
Oppositional defiant disorder	45
Borderline personality disorder	33
Major depressive disorder	31
Generalized anxiety disorder	23
Separation anxiety disorder	19
Conduct disorder	18
Disruptive mood dysregulation disorder	17
Social phobia	9
Post‐traumatic stress disorder	3
Panic disorder	1

### 2.2 Procedures

We used data from the baseline assessment of a longitudinal study in which dyads completed a series of parent–child interaction tasks (in order: two baseline tasks, pre‐conflict anticipation, and a conflict discussion), which were videotaped. RSA was recorded continuously, and subjective affect ratings were collected throughout the protocol. Youth and parent behavior was recorded and coded. All procedures were approved by the Human Research Protection Office and the Clinical and Translational Science Institute pediatric practice‐based research network. Youth and parents provided written informed consent and were compensated.

### 2.3 Dyadic Interaction Tasks

#### 2.3.1 Baseline Tasks

Two 2‐min “vanilla” baselines consisted of youth sitting silently across from their parents for two min and listening to parents read an article for two  min. “Vanilla” baselines are recommended as a useful comparison point in psychophysiological research as providing a resting period with exposure to noncritical elements of the key task that are likely to induce RSA reactivity (e.g., through vocalization or listening) to better isolate effects of task on RSA reactivity (Jennings et al. 1992). RSA across these epochs were averaged. Baseline RSA has been shown to predict emotional flexibility, reciprocity, and mutual positivity during mother–child interactions (Connell et al. 2011, 2015; Crowell et al. 2013) and was therefore included as a control variable.

#### 2.3.2 Conflict Anticipation and Conflict Discussion

Research assistants identified two topics of disagreement rated highly in terms of frequency (*M* = 5.23; SD = 1.13) and intensity (*M* = 3.99; SD = 0.92) by both members of the dyad independently. Ratings were collected on a 25‐item questionnaire of common areas of parent–child conflict that was adapted from Hetherington et al. (1992) for the current study (Supporting Information Appendix A lists the full questionnaire administered to participants), where respondents rated areas of conflict in terms of frequency (1 = *once in past month* to 6 = *more than once per day*) and severity (1 = *not at all bad* to 5 = *extremely bad*). Figure S1 displays average ratings of severity for each area of conflict. Parents and youth were then asked to sit quietly for 2 min and think about what they wanted to say (conflict anticipation), before discussing the topic for 8 min to try to reach a possible solution (conflict discussion).

### 2.4 Multimodal Measurement

#### 2.4.1 Primary Outcome: Observed Youth Affective Behavior

Conflict discussions were videotaped and coded after the fact using a macro‐level coding manual adapted from the System for Coding Interactions and Family Functioning (SCIFF) (Lindahl and Malik 2000) and the Parenting Styles Rating Manual (Cowan and Cowan 1992). All codes are described in Table S1, with youth codes focused solely on youth behavior and parent behavior coded separately (see below). Each dimension was scored using a 5‐point Likert scale (1 = *very low* to 5 = *high*) with ratings including information about both frequency and intensity across the entire conversation (e.g., low levels include fewer instances of a behavior that must also be low intensity). Overall 15% of recordings were double coded for reliability, estimated by ICCs. Youth behavior was coded for anger/frustration (ICC = 0.85), rejection/invalidation (ICC = 0.85), opposition/defiance (ICC = 0.86), sadness/distress (ICC = 0.90), positive affect (ICC = 0.70), withdrawal (ICC = 0.71), and effective individuation (ICC = 0.83). Table S2 includes descriptive statistics and intercorrelations between all child‐level codes. Principal component analysis with varimax rotation was used for dimension reduction, revealing three components: (1) **anger/defiance** (explaining 30.99% of variance; comprising anger/frustration, rejection/invalidation, and opposition/defiance); (2) **sadness/distress** (explaining 9.08% of variance; comprised of sadness/distress); and (3) **positive engagement** (explaining 25.58% of the variance; comprised of positive affect, withdrawal (negative loading), and effective individuation). Factor scores were extracted and used in primary analyses. Tables S3–S4 and Figure S2 list eigenvalues, factor loadings, and a scree plot for these analyses.

Physiological activity (RSA) was sampled at 500 Hz using MindWare mobile devices and BioLab software (MindWare Technologies Ltd.).

An electrocardiogram with disposable Ag/Ag‐Cl spot electrodes was positioned in a modified lead‐II configuration. Two trained scorers visually inspected each recoded waveform (Berntson et al. 1997) and manually corrected artifacts using Mindware HRV 3.1.4 software (MindWare Technologies Ltd.). The interbeat interval series was resampled in 250 ms intervals, linearly detrended, and tapered using a Hanning window. Heart rate variability was calculated using Fast Fourier transformation analysis, and high‐frequency heart rate variability associated with the log‐transformed high‐frequency respiratory power band (0.12–0.50 Hz range; Berntson et al. 1997) was used as a measure of RSA. Bandwidth of 0.12–0.50 Hz was selected on the basis of the initial inspection of the data revealing that some youths’ (*n* = 53) peak respiratory frequency fell at/above 0.40 Hz (the more typical upper limit). Peak respiration frequency in the full sample ranged from 0.19 to 0.50 across all tasks.

RSA was estimated separately in 1‐min intervals for the two baseline tasks, the 2‐min conflict anticipation, and the 8‐min conflict discussion. Because *n* = 8 dyads went off‐topic by the last minute of conflict and behavior after this point was not coded, only the first 7 min of conflict discussion was analyzed. RSA estimates for all 4 min of baseline tasks were averaged for a single measure of baseline RSA (*α* = 0.66). Individual RSA observations >3 SD outside the sample mean were removed, resulting in missing data for two subjects for 4 and 2 min of the conflict task, respectively. One‐min intervals for RSA estimation were chosen on the basis of recommendations (Camm et al. 1996; Laborde et al. 2017) stating that this interval length allows for a more reliable estimate of RSA.

#### 2.4.2 Youth Subjective Negative Affect

Prior to conflict anticipation and immediately following conflict discussion, youth completed the Positive and Negative Affect Schedule (PANAS; Watson et al. 1988) Short Form, consisting of 10 emotion items rated on a 1 (*very slightly* or *not at all*) to 5 (*extremely*) scale. Ratings of negative affect (sad, nervous, upset, mad, ashamed) were summed for a total score, with weak internal consistency pre‐anticipation (*α* = 0.58) but acceptable internal consistency post‐conflict (*α* = 0.79). Change scores were calculated by subtracting pre‐anticipation scores from post‐conflict scores. Higher scores indicated increases in negative affect.

#### 2.4.3 Observed Negative Parent Behavior

Using our adapted coding manual (see online supplement for greater detail), parents were coded on dimensions of positive affect (ICC = 0.80), anger/frustration (ICC = 0.84), rejection/invalidation (ICC = 0.82), coerciveness (ICC = 0.72), emotional support (ICC = 0.84), and respect for autonomy (ICC = 0.81). In a principal component analysis, all dimensions loaded onto a single factor representing negative parent behavior. This factor explained 59.66% of variance and was extracted for analysis. The online supplement includes descriptive statistics and intercorrelations (Table S2) between codes in addition to the eigenvalues (Table S5), factor loadings (Table S6), and a scree plot (Figure S3) for principal component analysis.

#### 2.4.4 Additional Covariates

Youth age, sex (0 = female; 1 = male), and same‐day stimulant medication use (0 = no stimulants; 1 = stimulant use) were included as potential covariates. However, upon examining bivariate correlations with all study variables, none were associated with both dependent (youth affective behavior) and independent (youth RSA) variables and thus were not included in final analyses.

### 2.5 Data Analytic Strategy

To characterize trajectories of youth RSA, we used latent growth curve modeling in Mplus 8.8 (Muthén and Muthén 1998) with FIML estimation. A piecewise growth model estimated two separate linear slopes of minute‐by‐minute change in RSA during conflict anticipation and conflict discussion. Prior to this, we compared multiple models to identify the most appropriate slope of RSA during conflict alone (Table S7; intercept‐only to cubic change models evaluated with a linear change model demonstrating best fit to the data). The piecewise model estimates a single intercept factor and two separate slopes, with the first minute of the conflict discussion serving as the inflection, or knot point, and included in both slopes. The intercept was set to the first minute of anticipation; however, supplemental analyses based on reviewer responses with the intercept set to the first minute of conflict yielded identical results. Next, latent factor scores for intercept and both slopes (conflict anticipation and conflict discussion) were extracted for further analysis, conducted in SPSS version 29 (IBM Corp. 2022). To predict the youth affective behavior (anger/defiance, sad/distress, positive engagement), we tested three separate regressions. Because the youth affective behavior variables were derived from a PCA, they were uncorrelated, which is why they were examined in separate models. Predictors were entered sequentially in three blocks: (1) youth baseline RSA, RSA intercept, and slope; (2) youth subjective negative affect; and (3) observed negative parent behavior.

## 3 Results

### 3.1 Preliminary Analyses

The piecewise latent growth model demonstrated good fit to the data (*χ* ^2^(36) = 39.47, *p* = 0.318; RMSEA = 0.25; CFI = 0.997). Table 2 includes the estimated mean and variance for the intercepts and slopes from this model. RSA withdrawal was observed during conflict anticipation and conflict discussion (see Figure S2); however, only conflict discussion slope was significant. To determine whether slopes were significantly different across these two periods, a contrast between slopes was tested, revealing no significant difference at the sample level (Est = −0.03, SE = 0.04, *p* = 0.946). Intercept and slope variance during conflict anticipation was significant, suggesting between‐person differences in levels of RSA in the first interval and within‐person differences in rate of RSA withdrawal during conflict anticipation.

**TABLE 2:** Estimates for piecewise growth curve model of minute‐by‐minute respiratory sinus arrythmia (RSA) in anticipation of and during parent–child conflict.

	Mean		Variance	
	*b* (SE)	*p*	*b* (SE)	*p*
Intercept	6.82 (0.09)	<0.001	1.14 (0.15)	<0.001
Anticipation slope	−0.02 (0.03)	0.464	0.10 (0.02)	<0.001
Conflict slope	−0.03 (0.01)	0.003	0.00 (0.00)	0.117

Table 3 shows bivariate correlations between all study constructs. RSA intercept was negatively associated with RSA slope during conflict anticipation, such that higher levels of RSA in the first interval were associated with faster RSA withdrawal during conflict anticipation. Steeper RSA withdrawal in conflict anticipation was associated with less steep withdrawal during conflict discussion. RSA slope during conflict anticipation was only related to observed negative parent behavior during conflict: Among youth whose parents expressed more negative behavior during conflict, their RSA withdrew more steeply when anticipating conflict. RSA slope during conflict discussion was only correlated with negative parent behavior, showing that for youth whose parents’ exhibited more negative behavior during conflict, their RSA withdrew more slowly.

**TABLE 3:** Correlations between and descriptive statistics of study variables.

	1.	2.	3.	4.	5.	6.	7.	8.	9.	10.	11.	12.
1. Youth sex (female)												
2. Youth age	0.25**											
3. Same day stimulant use	−0.25**	−0.10										
4. Baseline RSA	0.07	−0.05	−0.09									
5. RSA intercept	0.07	0.01	−0.09	0.88**								
6. RSA slope—anticipation	−0.02	−0.09	0.01	−0.19*	−0.47**							
7. RSA slope—conflict	−0.04	0.03	0.02	0.05	0.15	−0.40**						
8. Subjective youth negative affect (change)	0.06	−0.04	−0.17*	−0.12	−0.09	−0.08	0.18*					
9. Observed negative parent behavior	−0.01	−0.11	−0.03	0.17*	0.21**	−0.16	0.16*	0.20*				
10. Observed anger and defiance	0.11	0.03	−0.00	0.02	0.03	−0.01	−0.09	0.28**	0.26**			
11. Observed sadness and distress	−0.02	−0.03	−0.13	−0.10	−0.09	−0.06	0.19*	0.41**	0.14	0.00		
12. Observed positive engagement	0.13	0.08	−0.06	−0.14	−0.13	0.06	−0.12	−0.04	−0.51**	0.00	0.00	
Mean/% (SD)	46.9%	12.59 (0.95)	17.9%	6.63 (1.04)	6.81 (1.08)	−0.02 (0.26)	−0.02 (0.03)	1.15 (3.15)	0.00 (1.00)	0.00 (1.00)	0.00 (1.00)	0.00 (1.00)
Skew		−0.34		−0.23	−0.46	0.57	0.36	1.14	0.37	1.31	1.39	−0.32
Kurtosis		−1.00		1.39	1.67	1.54	1.13	2.29	−0.64	1.71	2.05	−0.35

*p<0.05.

**p<0.01.

We observed correlations between observed youth and parent behavior, with more negative parent behavior associated with more anger/defiance and less positive engagement by youth. More negative parent behavior was also associated with a stronger increase in negative affect through conflict. Greater increases in subjective negative affect by youth were also associated with more observed anger/defiance and sadness/distress during the conflict discussion.

### 3.2 Predicting Youth Affective Behavior During Conflict Anticipation and Conflict Discussion

Table 4 includes results from three separate regressions predicting observed youth affective behavior: *anger/defiance, sadness/distress*, and *positive engagement*.

**TABLE 4:** Regressions predicting observed youth behavior during parent–child conflict discussions.

	Block 1 Youth RSA				Block 2 Youth negative affect (change)				Block 3 Observed negative parent behavior			
**DV: Angry and defiant behavior**	*B* (SE)	*b*	*p*	*R* ^2^	*B* (SE)	*b*	*p*	Δ*R* ^2^	*B* (SE)	*b*	*p*	Δ*R* ^2^
Baseline RSA	−0.05 (0.21)	−0.05	0.812		0.00 (0.20)	0.00	0.994		−0.02 (0.20)	−0.02	0.922	
RSA intercept	0.11 (0.22)	0.11	0.611		0.09 (0.21)	0.09	0.675		0.07 (0.21)	0.07	0.730	
RSA slope—anticipation	−0.08 (0.47)	−0.02	0.871		−0.10 (0.45)	−0.03	0.824		−0.04 (0.44)	−0.01	0.937	
RSA slope—conflict	−4.16 (3.33)	−0.12	0.213	0.014	−5.94 (3.23)	−0.17	0.068		−**6.58 (3.18)**	**−0.18**	**0.041**	
Subjective youth negative affect					**0.10 (0.03)**	**0.30**	**<0.001**	0.087	**0.09 (0.03)**	**0.26**	**0.002**	
Observed negative parent behavior									**0.21 (0.08)**	**0.21**	**0.014**	0.039
**DV: Sadness and distress**	*B* (SE)	*b*	*p*	*R* ^2^	*B* (SE)	*B*			*B* (SE)	*b*		
Baseline RSA	0.04 (0.21)	0.04	0.843		0.11 (0.19)	0.10	0.574		0.10 (0.19)	0.09	0.604	
RSA intercept	−0.23 (0.23)	−0.22	0.305		−0.26 (0.21)	−0.25	0.208		−0.27 (0.21)	−0.26	0.196	
RSA slope—anticipation	−0.30 (0.47)	−0.08	0.523		−0.33 (0.43)	−0.09	0.445		−0.31 (0.43)	−0.08	0.482	
RSA slope—conflict	6.57 (3.35)	0.18	0.052	0.047	4.22 (3.13)	0.12	0.179		3.97 (3.14)	0.11	0.208	
Subjective youth negative affect					**0.13 (0.03)**	**0.39**	**<0.001**	0.146	**0.13 (0.03)**	**0.37**	**<0.001**	
Observed negative parent behavior									0.08 (0.08)	0.08	0.316	0.006
**DV: Positive engagement**	*B* (SE)	*b*	*p*	*R* ^2^	*B* (SE)	*b*			*B* (SE)	*b*		
Baseline RSA	−0.21 (0.21)	−0.20	0.312		−0.21 (0.21)	−0.20	0.305		−0.16 (0.18)	−0.15	0.371	
RSA intercept	0.09 (0.22)	0.09	0.695		0.09 (0.22)	0.09	0.689		0.13 (0.20)	0.13	0.494	
RSA slope—anticipation	0.19 (0.46)	0.05	0.689		0.19 (0.47)	0.05	0.687		0.03 (0.41)	0.01	0.949	
RSA slope—conflict	−3.65 (3.33)	−0.10	0.274	0.035	−3.51 (3.38)	−0.10	0.301		−1.89 (2.94)	−0.05	0.521	
Subjective youth negative affect					−0.01 (0.03)	−0.02	0.779	0.001	0.03 (0.03)	0.08	0.301	
Observed negative parent behavior									−**0.52 (0.08)**	−**0.52**	**<0.001**	0.242

*Note:* Dependent variables are shown in the left column corresponding with the three separate regressions run predicted by sets of variables added in three separate blocks.

*p *< 0.05.

*p *< 0.01.

For *anger/defiance*, no RSA indicators were statistically significant (Block 1). When adding subjective negative affect into the model (Block 2), RSA indicators remained non‐significant; however, youth subjective negative affect had a positive effect on observed anger/defiance, accounting for 9% additional variance explained. Once accounting for parents’ negative behavior (Block 3), the effect of RSA withdrawal during conflict discussion became significant, such that anger/defiance was associated with a faster rate of withdrawal. Additionally, both youth subjective negative affect and negative parent behavior (accounting for an additional 4% of variance) were positively associated with more youth anger/defiance.

In a series of sensitivity analyses (see Tables S8 and S9), we first re‐ran the model when using subjective negative affect as measured post‐conflict discussions (rather than the change score). Although the magnitude of effects was largely similar (*B*(SE) = −0.580 (3.13), *b* = −0.16, *p* = 0.066), the effect of RSA withdrawal during conflict was no longer statistically significant. We also re‐ran the model using individual ratings of each negative affect and found that overall pattern of results was the same; however, the effect of subjective negative affect was specifically driven by reports of feeling mad.

For *sadness/distress*, no RSA indicators were statistically significant (Block 1). Youth subjective negative affect had a significant and positive effect on youth sadness/distress, accounting for an additional 15% of variance. Including observed negative parent behavior in the model did not alter results (Block 3) and only accounted for an additional 1% of variance. Sensitivity analysis revealed the same pattern of findings when using subjective reports of negative affect from post‐conflict discussions (Table S8). Furthermore, effect of subjective negative affect was driven by ratings of sadness and shame, specifically (Table S10).

For *positive engagement*, there were no significant effects of RSA indicators in Block 1. In Block 2, RSA indicators remained non‐significant, and subjective negative affect was unrelated to positive engagement. In Block 3, negative parent behavior predicted less youth positive engagement, accounting for an additional 24% of the variance. Results from sensitivity analyses (Tables S8 and S11) revealed no change to the pattern of findings.

## 4 Discussion

The current study aimed to characterize associations between RSA activity and observed youth affective behavior during parent–child conflict. These effects were examined in a diverse clinical sample during the sensitive transition to adolescence, using a multimodal approach, which included assessment of youth subjective negative affect and observed negative parenting behavior. Our piecewise trajectory analyses demonstrated non‐significant RSA withdrawal during conflict anticipation and, in line with previous studies (Cui et al. 2015; Miller et al. 2013), significant RSA withdrawal during conflict discussion. This approach allows for an exploration of independent effects of RSA during different phases of conflict on youth observed affective behavior.

There was a unique negative association between the RSA slope during conflict discussion and observed youth *anger/defiance* that emerged only after controlling for youth subjective negative affect and observed negative parenting behavior. This is in line with other studies that have found associations between youth RSA withdrawal and negative affect expressions during challenging interactions (Crowell et al. 2014; Glackin et al. 2021). This highlights the importance of accounting for these other domains when examining affective response. Given that associations with RSA activity were observed only after holding constant perceptions of safety in the environment (Porges 2007), our results suggest that youths’ subjective experience, as well as objective measures of context, may have functioned as proxies for safety signals. Along these lines, we found positive correlations between observed youth *anger/defiance* and youth subjective negative affect and observed negative parent behavior. This echoes previous work showing that simultaneous elevations in subjective, observed, and physiological experience were linked to behavior dysregulation (Hubbard et al. 2004) and points to the importance of replicating these findings to determine whether a concordant, multimodal response is indeed what characterizes angry and defiant behavior within‐youth (Lanteigne et al. 2014).

It should be noted that sensitivity analyses testing whether similar effects were observed when using post‐conflict scores of subjective negative affect instead of difference scores revealed that RSA activity was no longer significant as a predictor of youth *anger/defiance*. This may mean that subjective negative affect post‐discussion (state level) was a stronger predictor relative to subjective emotional reactivity (difference scores)—explaining a greater proportion of variance. Difference scores have various noted limitations, including multicollinearity between difference scores and the original scores used to derive them as well as statistical noise that can constrain associations with outcome variables (Laird and De Los Reyes 2013). These limitations of difference scores could have led to spurious significance or suppression effects in the primary models. For this reason, future research employing continuous scores of subjective affect could measure and model predictors on the same timescale for a greater understanding of the effects of state levels and reactivity of subjective affect in the association between physiology and behavior (more discussion on this below).

Less pronounced RSA withdrawal during conflict initially predicted observed *sadness/distress*, consistent with prior work showing RSA augmentation during expressions of sadness (Marsh et al. 2008). However, this association was fully accounted for by youths’ subjective negative affect. Youth reports of increases in negative affect were associated with more expressions of sadness/distress. The fact that there was no association between physiology and sad/distressed behavior in youth after accounting for subjective negative affect highlights the range of types and functions of sadness (e.g., anticipatory or acute sadness), which are associated with different patterns of physiological responding, including RSA activity (Kreibig et al. 2025). It could be that accounting for subjective affect cancelled out differential patterns of RSA response. Additionally, on the basis of the analysis of individual behavioral codes comprising each of our components used in analysis, our code for sadness/distress was not characterized by withdrawal and thus may reflect active expressions of sadness (e.g., crying), which has been found to not be associated with RSA activity among depressed participants and with increases in RSA among nondepressed participants (Rottenberg et al. 2003).

Lastly, *positive engagement*, operationalized as expressions of positive affect, effective individuation, and less withdrawal, was solely predicted by less observed negative parent behavior. This suggests that effective engagement by youth is not characterized by any single pattern of physiological or subjective experience. However, it highlights the importance of reciprocity between parent and child for effective conflict discussions. Notably, negative parent behavior accounted for an additional 24% of variance in youth positive engagement, whereas it only accounted for 1%–4% of additional variance in observed anger/defiance and sadness/distress. Research has long emphasized that successful individuation and autonomy development in adolescents are highly related to patterns of parent‐adolescent transactions (Branje 2018; Koepke and Denissen 2012). Although our research design did not allow us to model these reciprocal patterns, our findings are consistent with the possibility that positive engagement by youth was facilitated by positive parent behavior (Bülow et al. 2022).

The fact that RSA activity was uniquely associated with angry and defiant behavior, but not sadness/distress or positive engagement, is aligned with observations in different emotion‐induction tasks showing that RSA withdrawal is most pronounced during anger induction relative to sadness and happiness induction (Fortunato et al. 2013; Konopkina et al. 2024) and other research showing that physiological responding generally tracks only very modestly with other affective response domains (Mauss et al. 2005). However, RSA reactivity to anger induction, specifically, has been associated with lower emotion regulation skills over time (Gatzke‐Kopp et al. 2015), and greater RSA withdrawal during parent–child conflict has been associated with emotion dysregulation and externalizing forms of behavioral dysregulation in daily life among adolescents (Byrd et al. 2022). Our study provides greater clarity on physiology‐behavior associations during the transition to adolescence, when dysregulated emotion and behavior increase alongside risk for psychopathology (Cavicchioli et al. 2023). There have been documented developmental changes in RSA activity across childhood (Hinnant and El‐Sheikh 2009, 2013; Stephens et al. 2021) and into adolescence (Dollar et al. 2020) suggesting increased RSA reactivity. Thus, it is important to interpret findings in the context of the developmental phase we studied. It is possible that a similar magnitude of RSA withdrawal observed in earlier ages would predict greater variability in behavior, whereas this level of reactivity becomes more normative by this age and especially within a clinical sample. Future research should explore the developmental continuity of physiology‐behavior associations in the context of psychopathology development.

Considering the larger context of psychopathology development, models have suggested conceptualizing physiological activity as an adaptive stress response to early environments and rearing contexts (Ellis et al. 2017; Ellis and Giudice 2019). For example, the Adaptive Calibration Model posits that in response to stressful, high‐risk environments, a vigilant profile of physiological activity characterized by reciprocal increases in sympathetic and decreases in parasympathetic activity enables individuals to cope effectively with environmental threats. While adaptive for coping with high‐stress environments, this physiological profile is characterized behaviorally by both externalizing (especially in males) and internalizing (especially in females) pathology (Del Giudice et al. 2011, 2012), suggesting costs for emotional well‐being while still reflecting an adaptive response to early rearing environments. Considering our findings that a pronounced parasympathetic response may underlie angry and defiant behavior during conflict and extend findings that expressions of hostility toward parents predict greater externalizing pathology in adolescents (Martin et al. 2019), we propose a process by which this reactive physiological profile increases likelihood of developing psychopathology over time. More importantly, results of this article suggest that targeting parasympathetic reactivity in parent–child conflicts (see Linehan 1993 for one approach) has the potential to reduce conflict behaviors that could be damaging not only to the parent–child relationship but also to youths’ well‐being.

### 4.1 Limitations

Findings should be considered within the context of several limitations. First, we focused on parent–child conflict, given the developmental salience during this period; however, discussions designed to elicit sadness may be better suited to investigate individual differences in this domain of affective behavior. Second, although we focused on correlates of youth affective behavior immediately prior to and during conflict, youth affective behavior is also impacted by other factors, including youth disposition, history of parenting, and even frequency and quality of recent conflicts (Eisenberg et al. 2008). Future work should incorporate these factors. Importantly, we assessed physiological response during conflict anticipation and found that faster RSA withdrawal during conflict anticipation was associated with less sharp RSA withdrawal during conflict discussions, suggesting that youth show some signs of physiological preparation for anticipated conflict. However, our anticipation period was short and did not include a specific assessment of subjective negative affect. Finally, although we conducted a multimodal assessment of youth affective responses, both observed and subjective indicators were measured more statically was RSA. Thus, we were not able to evaluate the more dynamic changes within and across those domains. Future research could employ continuous coding of affective behaviors and use video‐mediated recall (e.g., Mudiam et al. 2023) to assess more momentary expressions and subjective experiences, respectively. Additionally, by measuring different modalities on the same timescale, future research could explore whether different modalities function as interacting systems to influence youth behavior, whereas the current study treated different modalities as parallel predictors. Furthermore, a larger sample would allow us to estimate nonlinear change in RSA as well as moderations with other variables of interest. Lastly, this study did not include an assessment of parent RSA; however, research has shown that parent–child RSA synchrony may represent biological sensitivity to context (e.g., Oshri et al. 2021, 2023) highlighting the importance of understanding the dynamics of RSA activity in both dyad members when studying the parent–child relationship and interactions.

Despite these limitations, this study represents a unique contribution to the field by capturing three domains of youths’ affective responding (observed behavior, physiology, subjective experience) in during a developmentally salient context during the transition to adolescence. Results identified distinct patterns of affective responses that predicted different forms of youth behavior. Future research could elaborate on these findings to understand how patterns of correspondence across different modes of behavior, using person‐centered analysis, predict outcomes for youth, both individually and in their relationships with parents into adolescence and adulthood.

## Funding

This research was supported by the National Institute of Mental Health with grants K01 MH131755 (to S.V.), K01 MH119216 (to A.L.B.), and R01 MH101088 (to S.D.S.), and Clinical and Translational Science Institute, University of Pittsburgh withgrant UL1 TR001857 (to S.D.S.).

## Ethics Statement

All procedures were approved by the Institutional Review Board at the University of Pittsburgh.

## Consent

All participants completed a signed informed consent.

## Conflicts of Interest

The authors declare no conflicts of interest.

## Supporting information




**Table S1**. Description of observational codes.
**Figure S1**. Mean severity ratings for conflict topics.
**Table S2**. Descriptive statistics and intercorrelations between observational codes.
**Table S3**. Eigenvalues and variance explained for principal component analysis on youth affective behavior.
**Figure S2**. Scree plot for principal component analysis on youth affective behavior.
**Table S4**. Component matrix for three extracted components of youth affective behavior.
**Table S5**. Eigenvalues and variance explained for principal component analysis on parent behavior.
**Figure S3**. Scree plot for principal component analysis on parent behavior.
**Table S6**. Component matrix for negative parent behavior.
**Table S7**. Model fit values for latent growth curve models estimating minute‐by‐minute RSA values during parent–child conflict discussion.
**Figure S4**. Sample and estimated means of minute‐by‐minute RSA in anticipation of and during parent–child conflict discussion.
**Table S8**. Regressions predicting observed youth behavior during parent–child conflict discussions, including subjective youth negative affect measured post‐conflict discussion.
**Table S9**. Regressions predicting observed youth angry and defiant behavior during parent–child conflict discussions, including individual reports of subjective negative affect.
**Table S10**. Regressions predicting observed youth sadness and distress behavior during parent–child conflict discussions, including individual reports of subjective negative affect.
**Table S11**. Regressions predicting observed youth positive engagement behavior during parent–child conflict discussions, including individual reports of subjective negative affect.
**Appendix A**. Hot topics form (child and parent version).

## Data Availability

The data necessary to reproduce the analyses presented here are not publicly accessible.
