# Supplementary material for: Investigating Physiology‐Behavior Associations for Youth During Parent–Child Conflict Discussions
Source: Dev Psychobiol. 2026 Mar 31;68(3):e70149. doi: 10.1002/dev.70149 (PMC13036589; doi:10.1002/dev.70149)
Supplement: Supplementary file 1 — Table S1. Description of observational codes. Figure S1. Mean severity ratings for conflict topics. Table S2. Descriptive statistics and intercorrelations between observational codes. Table S3. Eigenvalues and variance explained for principal component analysis on youth affective behavior. Figure S2. Scree plot for principal component analysis on youth affective behavior. Table S4. Component matrix for three extracted components of youth affective behavior. Table S5. Eigenvalues and variance explained for principal component analysis on parent behavior. Figure S3. Scree plot for principal component analysis on parent behavior. Table S6. Component matrix for negative parent behavior. Table S7. Model fit values for latent growth curve models estimating minute‐by‐minute RSA values during parent–child conflict discussion. Figure S4. Sample and estimated means of minute‐by‐minute RSA in anticipation of and during parent–child conflict discussion. Table S8. Regressions predicting observed youth behavior during parent–child conflict discussions, including subjective youth negative affect measured post‐conflict discussion. Table S9. Regressions predicting observed youth angry and defiant behavior during parent–child conflict discussions, including individual reports of subjective negative affect. Table S10. Regressions predicting observed youth sadness and distress behavior during parent–child conflict discussions, including individual reports of subjective negative affect. Table S11. Regressions predicting observed youth positive engagement behavior during parent–child conflict discussions, including individual reports of subjective negative affect. Appendix A. Hot topics form (child and parent version). [file DEV-68-e70149-s001.docx]

**Supplementary Materials**

Investigating multimodal affective responses associated with youth behavior during parent-child conflict discussions.

**Table of Contents**

1. **Table S1.** *Description of observational codes*
2. **Figure S1.** *Mean severity ratings for conflict topics*
3. **Table S2.** *Descriptive Statistics and Intercorrelations Between Observational Codes*
4. **Table S3.** *Eigenvalues and variance explained for principal component analysis on youth affective behavior*
5. **Figure S2.** *Scree plot for principal component analysis on youth affective behavior*
6. **Table S4.** *Component matrix for three extracted components of youth affective behavior*
7. **Table S5.** *Eigenvalues and variance explained for principal component analysis on parent behavior*
8. **Figure S3.** *Scree plot for principal component analysis on parent behavior*
9. **Table S6.** *Component matrix for negative parent behavior*
10. **Table S7.** *Model fit values for latent growth curve models estimating minute-by-minute RSA values during parent-child conflict discussion*.
11. **Figure S4.** *Sample and estimated means of minute-by-minute RSA in anticipation of and during parent-child conflict discussion.*
12. **Table S8.** *Regressions predicting observed youth behavior during parent-child conflict discussions, including Subjective Youth Negative Affect measured post-conflict discussion*.
13. **Table S9.** *Regressions predicting observed youth angry & defiant behavior during parent-child conflict discussions, including individual reports of subjective negative affect.*
14. **Table S10.** *Regressions predicting observed youth sadness & distress behavior during parent-child conflict discussions, including individual reports of subjective negative affect.*
15. **Table S11.** *Regressions predicting observed youth positive engagement behavior during parent-child conflict discussions, including individual reports of subjective negative affect.*
16. **Appendix A.** *Hot Topics Form (Child and Parent Version)*
17. **References**

**Table S1.** *Description of observational codes*

The observational coding system used in the current study was adapted from two previous coding manuals: the Parenting Styles Rating Manual (Cowan & Cowan, 1992) and the System for Coding Interactions & Family Functioning (Lindahl & Malik, 2000). Adaptations were made to fit the sample, with the goal of coding parent and child behavior separately using parallel codes (with developmentally appropriate specifications). All codes were rated on a scale from 1 (Very Low) to 5 (High) with considerations for both the frequency and intensity of behaviors. Below are descriptions of each code as listed in the coding manual.

| Code | Code Description |
| --- | --- |
| Positive Affect (applied to parent and child separately) | This code assesses the positiveness of the parent/adolescent’s tone of voice, facial expressions, and body language on a scale from little to no positive affect expressed to much positive affect expressed. Positive affect may be expressed through behaviors such as affection, genuine joking around/laughter, and genuine smiling. Tone of voice can be happy, excited, upbeat, or satisfied. Facial expressions include smiling, laughing, or looking relaxed. |
| Anger & Frustration  (applied to parent and child separately) | This code assesses the overall level of negative affect (e.g., anger, frustration, tension, and irritation) expressed by the parent/adolescent through tone of voice, facial expressions, and body language during the interaction. Consider what the parent/adolescent says as well as how he/she says it. In other words, parents/adolescents may express frustration or tension either through verbalizations (e.g., I hate talking about this), overt behavior (e.g., yelling, pouting, banging on chair), or emotional tone (e.g., whining, frustrated, impatient, irritated, or angry). |
| Sadness & Distress (applied to parent and child separately)  IRR for parent-level code below threshold and was not included in analysis. | This code is primarily an affect or emotional/behavioral code, though at times parents/adolescents may be observed to make statements of sadness. It assesses the overall quantity of sadness, sorrow, anguish, grief, pain, regret, and remorse displayed by each individual.  These emotions may be displayed in the following manner: facial expressions such as tearfulness, sad frowns, or pained expressions, or looking as if the individual is crying or about to cry. Body gestures observed in conjunction with other expressions of sadness (in order not to confuse them with other codes, such as Withdrawal), may include slumped shoulders, downcast head or eyes, wringing hands, wiping tears, or putting one’s head in one’s hands. |
| Rejection/Invalidation (applied to parent and child separately) | This is primarily a content code based on the frequency and intensity with which a parent/adolescent makes cruel, critical, insulting, blaming, unkind, rude, or insensitive statements to the individual. It also includes behaviors that are dismissive or ignoring of the other individual’s feelings. This code assesses the overall level of rejection and/or invalidation expressed by the parent/adolescent. Rejection and invalidation may also be expressed through emotional tone (e.g., sounding disgusted, dismissing, or condescending about a parent/adolescent’s complaint, behavior, or expression of emotion). Other signs of rejection and invalidation include putting the individual down in some way or directly telling the individual not to experience an emotion. When discussing a problem, the parent/adolescent may criticize the individual’s character, rather than focusing only on the behavior. Invalidation also involves minimizing the importance of, disregarding, denying, or dismissing the parent/adolescent’s feelings, needs, and opinions. It may involve ignoring their emotional state when the individual is visibly upset. |
| Withdrawal (applied to parent and child separately)  IRR for parent-level code below threshold and was not included in analysis. | This code assesses the degree to which a parent/adolescent actively removes him/herself from the interaction or avoids the interaction or discussion. The individual may evade the issue or may seem to pull him/herself out of the discussion. The parent/adolescent may seem to retreat into a shell, become detached, back off, or shut down, physically or emotionally (in other words, through body language, tone of voice, and/or attitude). The parent/adolescent may withdraw by becoming indifferent, nonchalant, disinterested, or unresponsive. |
| Coerciveness (parent only) | This is a content code that is based on the frequency with which a parent makes threatening or manipulative statements to the child or uses a threatening tone or body language with the child. Coerciveness represents aversive or unpleasant methods that a parent uses to direct or control the child’s behavior. Coerciveness refers to threatening, bullying, shaming, embarrassing, or manipulative behaviors used by the parent. Threatening or overly punitive statements such as, “I have absolutely had it with your behavior -- do not push me! I have had it!” “The next time you do that, you won’t like the punishment,” or, “If you’re going to act like a spoiled brat, you’re going to get treated like one.” A parent may manipulate, shame, or embarrass the child by saying things like, "Well, we would love to take you out to dinner more often, but we can't because of your behavior." In addition, setting up questions so that there is only one right answer (and the right answer is to agree with the parent) is also coercive. Parental threat may also be expressed by saying in a bullying or superior tone, "I make the rules, you follow them." Bullying can also take the form of harsh, repetitive commands or demands such as, “Look at me! Look here! Look at me when I am talking to you!” |
| Emotional Support (parent only) | This code assesses several aspects of the supportiveness of the parent-child relationship, including emotional support and affective attunement or sensitivity. Emotional support refers to the parent's ability to 1) recognize and 2) meet the child's emotional needs and provide comfort or reassurance. This can be done verbally or through actions. This code assesses how sensitive, or attuned, the parent is to the child's emotional state, needs, and perspective, and how well they modify the child’s behavior accordingly. Affective attunement can be displayed either verbally (e.g., I can tell this is really frustrating) or nonverbally (e.g., facial expression, tone of voice). |
| Parent’s Respect for Adolescent Autonomy (parent only) | Two possible dimensions:   1. Parent allows a range of autonomous expressions on the part of the adolescent, i.e., gives adolescent room to make the interaction more his/her own and put his/her own style into it. These actions are seen to be more passive methods to allow for autonomy. In general the parent allows the adolescent to have opinions and contribute to decision making in the conversation. A parent may disagree with an adolescent’s ideas and still foster autonomy—it all depends on how the parent responds to and considers the adolescent’s input. 2. Parent shows respect more actively. Parent recognizes the adolescent's assertions of autonomy and adolescent's perspec­tive in general, acknowledges them, and validates them in various ways. |
| Effective Individuation (child only) | Possible dimensions/qualities of effectively asserting autonomy/individuation efforts:  Adolescent expresses a range of autonomous expressions. In general, the adolescent expresses his/her opinions and contributes to decision making in the conversation. An adolescent may disagree with a parent’s ideas but does so in a respectful manner. The following are examples of adolescent's autonomous behaviors that represent effective attempts at individuation:   1. Explore his/her conversation options (content possibilities of various tasks). 2. Initiate interactions (regulate them if he/she desires). 3. Assert his/her desires, needs, wishes, etc. 4. Take credit for his/her own ideas and accomplishments, i.e., to "own" them. 5. Express his/her thoughts and feelings freely (must be respectful/appropriate). |
| Opposition/Defiance (child only) | This code assesses the degree to which the adolescent displays oppositional, defiant, or belligerent behavior. Included in this code are insulting, distracting, disrespectful, noncompliant, disobedient, argumentative, annoying, blaming, angry, or vindictive behaviors. If the adolescent blames others for his/her mistakes, deliberately does things to annoy others, seems touchy or easily annoyed, or swears or deliberately says things to hurt others, the adolescent will be coded as high on the opposition/defiance code. |

**Figure S1.** *Mean severity ratings for conflict topics*

*
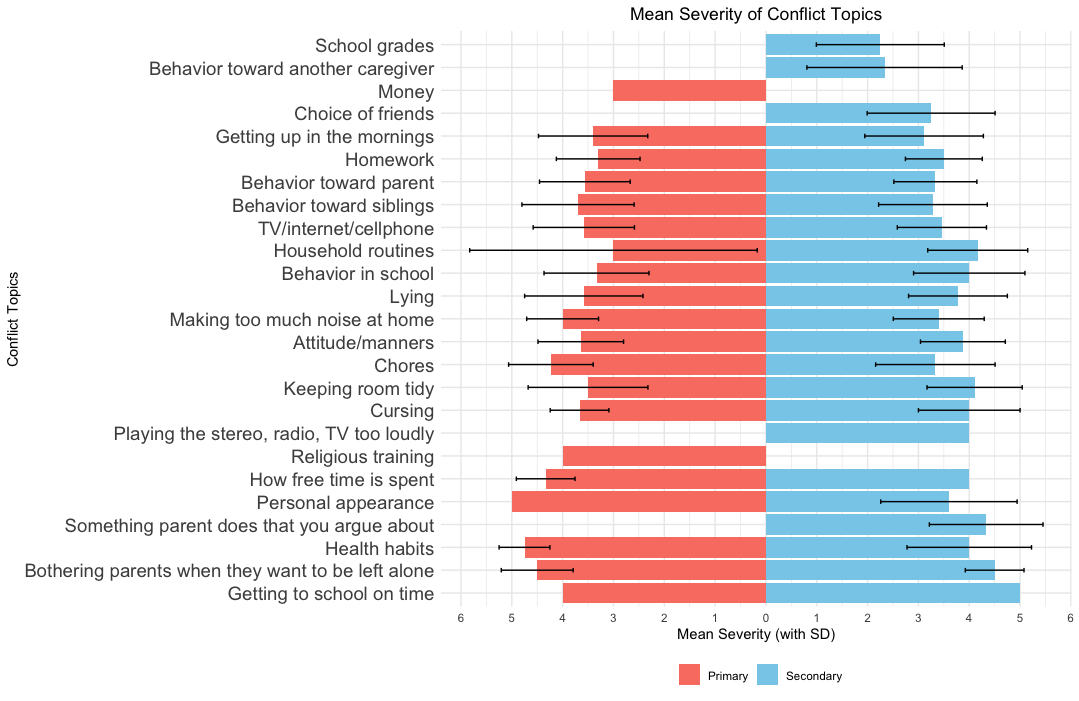
*

*Note*. This graph is reproduced from the supplementary materials from Thambipillai, Vine et al.) and depicts the mean severity ratings for various conflict topics reported by participants with a comparison between primary and secondary topics, with error bars indicating SD. Cases without error bars indicate topics identified by only one dyad. The severity ratings are based on a 5-point Likert scale (1= “not at all bad” to 5 = “extremely bad”)

**Table S2.** *Descriptive Statistics and Intercorrelations Between Observational Codes*

|  |  | 1 | 2 | 3 | 4 | 5 | 6 | 7 | 8 | 9 | 10 | 11 | 12 | 13 |
| --- | --- | --- | --- | --- | --- | --- | --- | --- | --- | --- | --- | --- | --- | --- |
| **PARENT CODES** | 1. Positive Affect |  |  |  |  |  |  |  |  |  |  |  |  |  |
|  | 2. Anger/Frustration | -.32** |  |  |  |  |  |  |  |  |  |  |  |  |
|  | 3. Rejection/Invalidation | -.31** | .68** |  |  |  |  |  |  |  |  |  |  |  |
|  | 4. Coerciveness | -.27** | .54** | .50** |  |  |  |  |  |  |  |  |  |  |
|  | 5. Emotional Support | .50** | -.60** | -.64** | -.50** |  |  |  |  |  |  |  |  |  |
|  | 6. Respect for Autonomy | .45** | -.49** | -.52** | -.45** | .75** |  |  |  |  |  |  |  |  |
| **CHILD CODES** | 7. Positive Affect | .63** | -.10 | -.10 | -.15 | .25** | .28** |  |  |  |  |  |  |  |
|  | 8. Anger/Frustration | -.06 | .34** | .18* | .05 | -.15 | -.07 | -.08 |  |  |  |  |  |  |
|  | 9. Sadness/Distress | -.19* | .13 | .19* | .21** | -.15 | -.24** | -.21** | .20* |  |  |  |  |  |
|  | 10. Rejection/Invalidation | -.04 | .14 | .11 | .04 | -.08 | -.01 | -.09 | .52** | .08 |  |  |  |  |
|  | 11. Withdrawal | -.35** | .24** | .19* | .20* | -.29** | -.28** | -.37** | -.09 | .15 | -.14 |  |  |  |
|  | 12. Effective Individuation | .40** | -.35** | -.34** | -.33** | .54** | .52** | .26** | -.10 | -.18* | -.15 | -.54** |  |  |
|  | 13. Opposition/Defiance | -.08 | .54** | .36** | .15 | -.32** | -.19* | .01 | .59** | -.07 | .41** | .02 | -.26** |  |
|  |  |  |  |  |  |  |  |  |  |  |  |  |  |  |
|  | Mean (SD) | 1.94 (1.17) | 2.20 (1.19) | 2.54 (1.29) | 1.81 (1.09) | 2.94 (1.13) | 3.41 (1.06) | 2.16 (1.30) | 2.13 (1.11) | 1.53 (1.12) | 1.34 (0.68) | 2.49 (1.20) | 2.79 (0.97) | 2.12 (1.23) |
|  | Skew | 1.11 | 0.75 | 0.56 | 1.48 | 0.13 | -0.38 | 0.82 | 0.94 | 2.25 | 2.76 | 0.45 | 0.13 | 0.81 |
|  | Kurtosis | 0.35 | -0.47 | -0.67 | 1.57 | -0.93 | -0.47 | -0.49 | 0.45 | 4.00 | 10.20 | -0.60 | -0.16 | -0.46 |

*Note.* **p*<.05, ***p*<.01. Observed range of scores was 1-5 for all codes.

**Table S3.** *Eigenvalues and variance explained for principal component analysis on youth affective behavior*

| Component | Total | % of Variance | Cumulative % |
| --- | --- | --- | --- |
| 1 | 2.17 | 30.99 | 30.99 |
| 2 | 1.79 | 25.58 | 56.57 |
| 3 | 1.02 | 14.61 | 71.18 |
| 4 | 0.76 | 10.87 | 82.05 |
| 5 | 0.57 | 8.12 | 90.17 |
| 6 | 0.39 | 5.52 | 95.69 |
| 7 | 0.30 | 4.31 | 100.00 |

**Figure S2.** *Scree plot for principal component analysis on youth affective behavior*


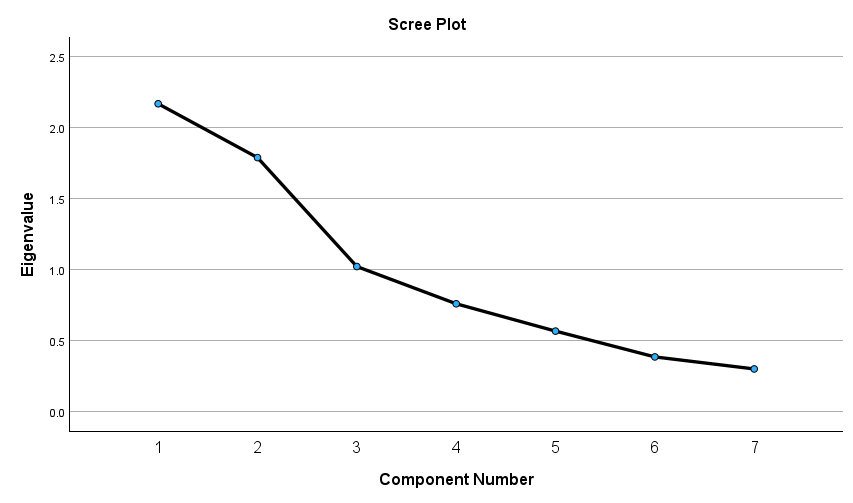


**Table S4.** *Component matrix for three extracted components of youth affective behavior*

|  | 1: Anger/Defiance | 2: Positive Engagement | 3: Sadness/Distress |
| --- | --- | --- | --- |
| Positive Affect | -.363 | .551 | -.240 |
| Anger/Frustration | .760 | .411 | .164 |
| Sadness/Distress | .311 | -.314 | .780 |
| Rejection/Invalidation | .670 | .397 | .110 |
| Withdrawal | .286 | -.795 | -.272 |
| Effective Individuation | -.560 | .552 | .328 |
| Opposition/Defiance | .720 | .356 | -.370 |

**Table S5.** *Eigenvalues and variance explained for principal component analysis on parent behavior*

| Component | Total | % of Variance | Cumulative % |
| --- | --- | --- | --- |
| 1 | 3.58 | 59.66 | 59.66 |
| 2 | 0.84 | 14.07 | 73.72 |
| 3 | 0.55 | 9.08 | 82.81 |
| 4 | 0.50 | 8.31 | 91.12 |
| 5 | 0.32 | 5.34 | 96.46 |
| 6 | 0.21 | 3.54 | 100.00 |

**Figure S3.** *Scree plot for principal component analysis on parent behavior*


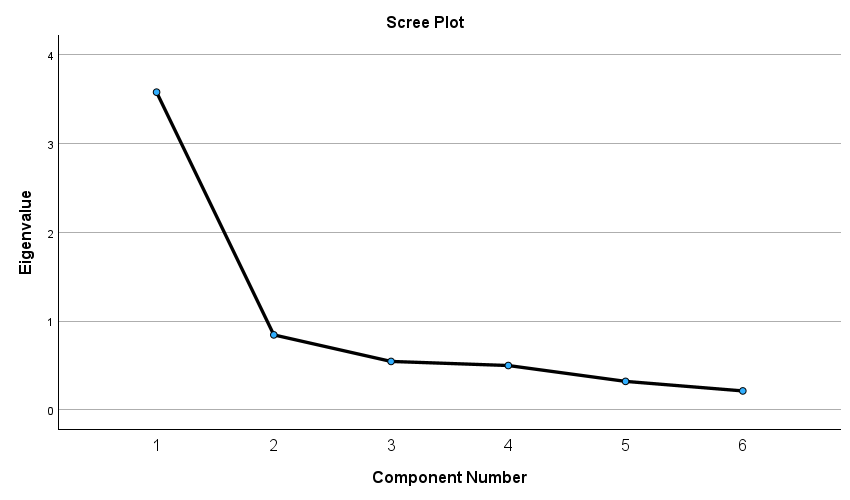


**Table S6.** *Component matrix for negative parent behavior*

|  | 1: Negative behavior |
| --- | --- |
| Positive Affect | -.589 |
| Anger/Frustration | .802 |
| Rejection/Invalidation | .810 |
| Coerciveness | .704 |
| Emotional Support | -.887 |
| Respect for Autonomy | -.806 |
| Positive Affect | -.589 |

**Table S7.** *Model fit values for latent growth curve models estimating minute-by-minute RSA values during parent-child conflict discussion.*

|  | *χ*^2^ | df | *p* | RMSEA | CFI | Comparison | Scaled difference | df – difference | *p* – difference |
| --- | --- | --- | --- | --- | --- | --- | --- | --- | --- |
| Intercept | 32.76 | 26 | .167 | 0.04 | 0.99 |  |  |  |  |
| Linear | 23.09 | 23 | .455 | 0.01 | 1.00 | v. Intercept | 8.897 | 3 | .031 |
| Quadratic | 20.72 | 22 | .538 | 0.00 | 1.00 | v. Linear | 2.894 | 1 | .089 |

*Note.* To evaluate the most appropriate shape of minute-by-minute change in RSA over the course of the conflict discussion, multiple models were tested and compared based on their model fit, especially given prior research demonstrating nonlinear change in RSA during parent-child conflict. Specifically, we estimated a no-growth (intercept only) model as well as linear, quadratic, and cubic growth curves. Fit indices evaluated included *χ^2^*, Comparative fit index (CFI; values higher than .95 deemed good fit; Bentler, 1990), and the root mean square error of approximation (RMSEA; values under .06 deemed good fit; Browne & Cudeck, 1993). Tests of *χ*^2^ difference (with scaling corrections for MLR) were used to compare nested models. *p*-values for comparisons < .05 interpreted to reflect statistically significant change in model fit.

Model fit was improved in a linear slope model compared to an intercept-only model, suggesting there was indeed change observed in RSA throughout the conflict discussion. When estimating a quadratic growth curve, the latent variable covariance matrix was non-positive-definite. Variance for the linear slope value was negative, but not statistically significant, so we fixed the estimate to 0 to allow the model to be estimated. However, model fit was not significantly improved compared to the linear model. The cubic model was not identified. We concluded that a linear growth curve model represented the best fit to the data.

**Figure S4.** *Sample and estimated means of minute-by-minute RSA in anticipation of and during parent-child conflict discussion.*

**Table S8.** Regressions predicting observed youth behavior during parent-child conflict discussions, including subjective youth negative affect measured *post-conflict discussion*.

|  | **Block 1** |  |  |  |  | **Block 2** |  |  |  |  | **Block 3** |  |  |  |
| --- | --- | --- | --- | --- | --- | --- | --- | --- | --- | --- | --- | --- | --- | --- |
|  | **Youth RSA** | | | |  | **Youth Negative Affect (post-conflict)** | | | |  | **Observed Negative Parent Behavior** | | | |
| **DV: Angry & Defiant Behavior** | B (SE) | *b* | *p* | *R^2^* |  | B (SE) | *b* | *p* | *ΔR^2^* |  | B (SE) | *b* | *p* | *ΔR^2^* |
| Baseline RSA | -0.06 (0.21) | -.06 | .764 |  |  | -0.05 (0.20) | -.04 | .821 |  |  | -0.06 (0.19) | -.06 | .758 |  |
| RSA Intercept | 0.13 (0.22) | .13 | .551 |  |  | 0.14 (0.21) | .14 | .515 |  |  | 0.12 (0.21) | .11 | .581 |  |
| RSA Slope - Anticipation | -0.07 (0.46) | -.02 | .874 |  |  | -0.03 (0.44) | -.01 | .955 |  |  | 0.03 (0.44) | .01 | .944 |  |
| RSA Slope - Conflict | -3.93 (3.31) | -.11 | .237 | .014 |  | -5.02 (3.18) | -.14 | .116 |  |  | -5.80 (3.13) | -.16 | .066 |  |
| Subjective Youth Negative Affect |  |  |  |  |  | **0.08 (0.02)** | **.30** | **<.001** | .089 |  | **0.07 (0.02)** | **.26** | **.002** |  |
| Observed Negative Parent Behavior |  |  |  |  |  |  |  |  |  |  | **0.21 (0.08)** | **.21** | **.012** | .040 |
|  |  |  |  |  |  |  |  |  |  |  |  |  |  |  |
| **DV: Sadness & Distress** | B (SE) | *b* | *p* | *R^2^* |  | B (SE) | *B* | *p* |  |  | B (SE) | *b* | *p* |  |
| Baseline RSA | 0.05 (0.21) | .04 | .826 |  |  | 0.07 (0.19) | .06 | .712 |  |  | 0.06 (0.19) | .06 | .736 |  |
| RSA Intercept | -0.24 (0.22) | -.23 | .288 |  |  | -0.23 (0.20) | -.22 | .260 |  |  | -0.24 (0.20) | -.23 | .242 |  |
| RSA Slope - Anticipation | -0.30 (0.47) | -.08 | .521 |  |  | -0.23 (0.43) | -.06 | .586 |  |  | -0.21 (0.43) | -.05 | .622 |  |
| RSA Slope - Conflict | 6.50 (3.32) | .18 | .052 | .047 |  | 4.97 (3.04) | .14 | .104 |  |  | 4.67 (3.05) | .13 | .129 |  |
| Subjective Youth Negative Affect |  |  |  |  |  | **0.12 (0.02)** | **.41** | **<.001** | .167 |  | **0.11 (0.02)** | **.40** | **<.001** |  |
| Observed Negative Parent Behavior |  |  |  |  |  |  |  |  |  |  | 0.08 (0.08) | .08 | .309 | .006 |
|  |  |  |  |  |  |  |  |  |  |  |  |  |  |  |
| **DV: Positive Engagement** | B (SE) | *b* | *p* | *R^2^* |  | B (SE) | *b* | *p* |  |  | B (SE) | *b* | *p* |  |
| Baseline RSA | -0.20 (0.21) | -.19 | .331 |  |  | -0.20 (0.21) | -.19 | .331 |  |  | -0.16 (0.18) | -.15 | .362 |  |
| RSA Intercept | -0.07 (0.22) | .07 | .738 |  |  | 0.07 (0.22) | .07 | .740 |  |  | 0.13 (0.19) | .13 | .496 |  |
| RSA Slope - Anticipation | 0.19 (0.46) | .05 | .691 |  |  | 0.18 (0.47) | .05 | .694 |  |  | 0.05 (0.40) | .01 | .912 |  |
| RSA Slope - Conflict | -3.82 (3.30) | -.11 | .249 | .038 |  | -3.79 (3.33) | -.11 | .256 |  |  | -1.86 (2.90) | -.05 | .522 |  |
| Subjective Youth Negative Affect |  |  |  |  |  | -0.00 (0.02) | -.01 | .917 | .000 |  | 0.02 (0.02) | .09 | .253 |  |
| Observed Negative Parent Behavior |  |  |  |  |  |  |  |  |  |  | **-0.53 (0.08)** | **-.52** | **<.001** | .243 |

Note. **p*<.05, ***p*<.01. Dependent variables are shown in the left column corresponding with the three separate regressions run predicted by sets of variables added in three separate blocks.

**Table S9.** Regressions predicting observed youth angry & defiant behavior during parent-child conflict discussions, including individual reports of subjective negative affect.

|  | **Block 1** |  |  |  |  | **Block 2** |  |  |  |  | **Block 3** |  |  |  |
| --- | --- | --- | --- | --- | --- | --- | --- | --- | --- | --- | --- | --- | --- | --- |
|  | **Youth RSA** | | | |  | **Youth Negative Affect (change)** | | | |  | **Observed Negative Parent Behavior** | | | |
|  | B (SE) | *b* | *p* | *R^2^* |  | B (SE) | *b* | *p* | *ΔR^2^* |  | B (SE) | *b* | *p* | *ΔR^2^* |
| Baseline RSA | -0.05 (0.21) | -.05 | .809 |  |  | -0.06 (0.19) | -.05 | .776 |  |  | -0.07 (0.19) | -.06 | .722 |  |
| RSA Intercept | 0.12 (0.23) | .11 | .613 |  |  | 0.09 (0.21) | .09 | .671 |  |  | 0.07 (0.21) | .07 | .726 |  |
| RSA Slope - Anticipation | -0.08 (0.47) | -.02 | .865 |  |  | -0.22 (0.44) | -.06 | .615 |  |  | -0.15 (0.44) | -.04 | .734 |  |
| RSA Slope - Conflict | -4.17 (3.36) | -.12 | .217 | .014 |  | **-6.75 (3.19)** | **-.19** | **.036** |  |  | **-6.90 (3.14)** | **-.19** | **.030** |  |
| Subjective Youth Sadness |  |  |  |  |  | 0.05 (0.12) | .04 | .710 |  |  | -0.01 (0.12) | -.01 | .914 |  |
| Subjective Youth Nervousness |  |  |  |  |  | -0.08 (0.08) | -.08 | .342 |  |  | -0.05 (0.08) | -.06 | .495 |  |
| Subjective Youth Upset |  |  |  |  |  | -0.05 (0.14) | -.05 | .693 |  |  | -0.06 (0.13) | -.06 | .637 |  |
| Subjective Youth Mad |  |  |  |  |  | **0.46 (0.12)** | **.45** | **<.001** |  |  | **0.46 (0.12)** | **.45** | **<.001** |  |
| Subjective Youth Shame |  |  |  |  |  | -0.04 (0.11) | -.04 | .710 | .195 |  | -0.03 (0.11) | -.03 | .771 |  |
| Observed Negative Parent Behavior |  |  |  |  |  |  |  |  |  |  | **0.18 (0.08)** | **.18** | **.033** | .027 |

Note. **p*<.05, ***p*<.01. Dependent variables are shown in the left column corresponding with the three separate regressions run predicted by sets of variables added in three separate blocks. When using individual reports of subjective negative affects from post-conflict (versus change scores), results were mostly unchanged; however, effect of slope of RSA during conflict discussions was slightly reduced in the final model (B(SE) = -6.25 (3.16), *b* = -.18, *p* = .050).

**Table S10.** Regressions predicting observed youth sadness & distress behavior during parent-child conflict discussions, including individual reports of subjective negative affect.

|  | **Block 1** |  |  |  |  | **Block 2** |  |  |  |  | **Block 3** |  |  |  |
| --- | --- | --- | --- | --- | --- | --- | --- | --- | --- | --- | --- | --- | --- | --- |
|  | **Youth RSA** | | | |  | **Youth Negative Affect (change)** | | | |  | **Observed Negative Parent Behavior** | | | |
|  | B (SE) | *b* | *p* | *R^2^* |  | B (SE) | *b* | *p* | *ΔR^2^* |  | B (SE) | *b* | *p* | *ΔR^2^* |
| Baseline RSA | -0.00 (0.20) | -.00 | .991 |  |  | 0.03 (0.18) | .02 | .890 |  |  | 0.02 (0.18) | .02 | .913 |  |
| RSA Intercept | -0.18 (0.22) | -.18 | .422 |  |  | -0.16 (0.20) | -.16 | .427 |  |  | -0.17 (0.20) | -.16 | .409 |  |
| RSA Slope - Anticipation | -0.14 (0.46) | -.04 | .766 |  |  | 0.01 (0.42) | .00 | .973 |  |  | 0.04 (0.42) | .01 | .917 |  |
| RSA Slope - Conflict | **7.31 (3.27)** | **.21** | **.027** | .051 |  | 4.55 (3.03) | .13 | .136 |  |  | 4.49 (3.03) | .13 | .142 |  |
| Subjective Youth Sadness |  |  |  |  |  | **0.24 (0.12)** | **.22** | **.043** |  |  | 0.22 (0.12) | .19 | .075 |  |
| Subjective Youth Nervousness |  |  |  |  |  | 0.01 (0.07) | .02 | .848 |  |  | 0.02 (0.08) | .02 | .760 |  |
| Subjective Youth Upset |  |  |  |  |  | 0.08 (0.13) | .07 | .543 |  |  | 0.08 (0.31) | .07 | .563 |  |
| Subjective Youth Mad |  |  |  |  |  | -0.03 (0.11) | -.03 | .824 |  |  | -0.03 (0.11) | -.03 | .827 |  |
| Subjective Youth Shame |  |  |  |  |  | **0.33 (0.10)** | **.30** | **.002** | .220 |  | **0.34 (0.10)** | **.30** | **.001** |  |
| Observed Negative Parent Behavior |  |  |  |  |  |  |  |  |  |  | 0.07 (0.08) | .07 | .366 | .005 |

Note. **p*<.05, ***p*<.01. Dependent variables are shown in the left column corresponding with the three separate regressions run predicted by sets of variables added in three separate blocks. When using individual reports of subjective negative affects from post-conflict (versus change scores), results were unchanged in the final model.

**Table S11.** Regressions predicting observed youth positive engagement behavior during parent-child conflict discussions, including individual reports of subjective negative affect.

|  | **Block 1** |  |  |  |  | **Block 2** |  |  |  |  | **Block 3** |  |  |  |
| --- | --- | --- | --- | --- | --- | --- | --- | --- | --- | --- | --- | --- | --- | --- |
|  | **Youth RSA** | | | |  | **Youth Negative Affect (change)** | | | |  | **Observed Negative Parent Behavior** | | | |
|  | B (SE) | *b* | *p* | *R^2^* |  | B (SE) | *b* | *p* | *ΔR^2^* |  | B (SE) | *b* | *p* | *ΔR^2^* |
| Baseline RSA | -0.20 (0.21) | -.19 | .341 |  |  | -0.21 (0.21) | -.20 | .321 |  |  | -0.17 (0.18) | -.16 | .351 |  |
| RSA Intercept | 0.08 (0.23) | .08 | .719 |  |  | 0.08 (0.23) | .08 | .720 |  |  | 0.13 (0.20) | .13 | .514 |  |
| RSA Slope - Anticipation | 0.20 (0.47) | .05 | .673 |  |  | 0.27 (0.49) | .07 | .587 |  |  | 0.05 (0.42) | .01 | .909 |  |
| RSA Slope - Conflict | -3.66 (3.35) | -.10 | .277 | .034 |  | -2.15 (3.48) | -.06 | .538 |  |  | -1.72 (3.04) | -.05 | .573 |  |
| Subjective Youth Sadness |  |  |  |  |  | -0.24 (0.13) | -.21 | .080 |  |  | -0.06 (0.12) | -.06 | .594 |  |
| Subjective Youth Nervousness |  |  |  |  |  | 0.03 (0.09) | .03 | .710 |  |  | -0.03 (0.08) | -.03 | .683 |  |
| Subjective Youth Upset |  |  |  |  |  | -0.11 (0.15) | -.09 | .483 |  |  | -0.08 (0.13) | -.07 | .557 |  |
| Subjective Youth Mad |  |  |  |  |  | 0.19 (0.13) | .19 | .152 |  |  | 0.19 (0.11) | .18 | .108 |  |
| Subjective Youth Shame |  |  |  |  |  | 0.11 (0.12) | .10 | .342 | .033 |  | 0.09 (0.10) | .08 | .405 |  |
| Observed Negative Parent Behavior |  |  |  |  |  |  |  |  |  |  | **-0.53 (0.08)** | **-.53** | **<.001** | .230 |

Note. **p*<.05, ***p*<.01. Dependent variables are shown in the left column corresponding with the three separate regressions run predicted by sets of variables added in three separate blocks. When using individual reports of subjective negative affects from post-conflict (versus change scores), results were unchanged in the final model.

**Appendix A.** *Hot Topics Form (Youth and Parent Version)*

| In the past month have you and your parent disagreed over any of these issues? (circle YES or NO) | | | IF YES, how often have you disagreed? (circle one) | | | | | | IF YES, how **bad** are the disagreement(s)?  (circle one) | | | | |
| --- | --- | --- | --- | --- | --- | --- | --- | --- | --- | --- | --- | --- | --- |
| 1. Your behavior toward your parent | yes | no | 1+ daily | once daily | 5-6x week | 3-4x week | 1-2x week | once a month | Extremely | very | somewhat | a little | not at all |
| 2. Your behavior toward another caregiver | yes | no | 1+ daily | once daily | 5-6x week | 3-4x week | 1-2x week | once a month | Extremely | very | somewhat | a little | not at all |
| 3. Your behavior toward your (step) brothers and sisters | yes | no | 1+  daily | once daily | 5-6x week | 3-4x week | 1-2x week | once a month | Extremely | very | somewhat | a little | not at all |
| 4. Your attitude/manners | yes | no | 1+  daily | once daily | 5-6x week | 3-4x week | 1-2x week | once a month | Extremely | very | somewhat | a little | not at all |
| 5. Your getting up in the mornings | yes | no | 1+ daily | once daily | 5-6x week | 3-4x week | 1-2x week | once a month | Extremely | very | somewhat | a little | not at all |
| 6. Your getting to school on time | yes | no | 1+ daily | once daily | 5-6x week | 3-4x week | 1-2x week | once a month | Extremely | very | somewhat | a little | not at all |
| 7. TV/internet/cell phone | yes | no | 1+ daily | once daily | 5-6x week | 3-4x week | 1-2x week | once a month | Extremely | very | somewhat | a little | not at all |
| 8. Your lying | yes | no | 1+ daily | once daily | 5-6x week | 3-4x week | 1-2x week | once a month | Extremely | very | somewhat | a little | not at all |
| 9. Your chores (what they are, how they should be done, etc.) | yes | no | 1+ daily | once daily | 5-6x week | 3-4x week | 1-2x week | once a month | Extremely | very | somewhat | a little | not at all |
| 10. Your health habits (eating, cleanliness, brushing teeth, etc.) | yes | no | 1+ daily | once daily | 5-6x week | 3-4x week | 1-2x week | once a month | Extremely | very | somewhat | a little | not at all |
| 11. Your religious training | yes | no | 1+ daily | once daily | 5-6x week | 3-4x week | 1-2x week | once a month | Extremely | very | somewhat | a little | not at all |
| 12. Your money (how much allowance, how money is spent) | yes | no | 1+ daily | once daily | 5-6x week | 3-4x week | 1-2x week | once a month | Extremely | very | somewhat | a little | not at all |
| 13. Your cursing | yes | no | 1+ daily | once daily | 5-6x week | 3-4x week | 1-2x week | once a month | Extremely | very | somewhat | a little | not at all |

| In the past month have you and your parent disagreed over any of these issues? (circle YES or NO) | | | IF YES, how often have you disagreed?  (circle one) | | | | | | IF YES, how **bad** are the disagreement(s)?  (circle one) | | | | |
| --- | --- | --- | --- | --- | --- | --- | --- | --- | --- | --- | --- | --- | --- |
| 14. Your personal appearance (choice of clothes, haircuts, etc.) | yes | no | 1+ daily | once daily | 5-6x week | 3-4x week | 1-2x week | once a month | Extremely | very | somewhat | a little | not at all |
| 15. Your household routines (bedtime, mealtimes, etc. ) | yes | no | 1+ daily | once daily | 5-6x week | 3-4x week | 1-2x week | once a month | Extremely | very | somewhat | a little | not at all |
| 16. Your homework (completing homework, quality of homework) | yes | no | 1+  daily | once daily | 5-6x week | 3-4x week | 1-2x week | once a month | Extremely | very | somewhat | a little | not at all |
| 17. Your school grades | yes | no | 1+  daily | once daily | 5-6x week | 3-4x week | 1-2x week | once a month | Extremely | very | somewhat | a little | not at all |
| 18. Your behavior in school | yes | no | 1+ daily | once daily | 5-6x week | 3-4x week | 1-2x week | once a month | Extremely | very | somewhat | a little | not at all |
| 19. Your making too much noise at home | yes | no | 1+ daily | once daily | 5-6x week | 3-4x week | 1-2x week | once a month | Extremely | very | somewhat | a little | not at all |
| 20. Your playing the stereo, radio, TV too loudly | yes | no | 1+ daily | once daily | 5-6x week | 3-4x week | 1-2x week | once a month | Extremely | very | somewhat | a little | not at all |
| 21. How your free time is spent | yes | no | 1+ daily | once daily | 5-6x week | 3-4x week | 1-2x week | once a month | Extremely | very | somewhat | a little | not at all |
| 22. Your choice of friends | yes | no | 1+ daily | once daily | 5-6x week | 3-4x week | 1-2x week | once a month | Extremely | very | somewhat | a little | not at all |
| 23. Keeping your room tidy | yes | no | 1+ daily | once daily | 5-6x week | 3-4x week | 1-2x week | once a month | Extremely | very | somewhat | a little | not at all |
| 24. Bothering your parents when they want to be left alone | yes | no | 1+ daily | once daily | 5-6x week | 3-4x week | 1-2x week | once a month | Extremely | very | somewhat | a little | not at all |
| 25. Something your parent does that you argue about. | yes | no | 1+ daily | once daily | 5-6x week | 3-4x week | 1-2x week | once a month | Extremely | very | somewhat | a little | not at all |

| In the past month have you and your child disagreed over any of these issues? (circle YES or NO) | | | IF YES, how often have you disagreed?  (circle one) | | | | | | IF YES, how **bad** are the disagreement(s)?  (circle one) | | | | |
| --- | --- | --- | --- | --- | --- | --- | --- | --- | --- | --- | --- | --- | --- |
| 1. His/her behavior toward you | yes | no | 1+ daily | once daily | 5-6x week | 3-4x week | 1-2x week | once a month | Extremely | very | somewhat | a little | not at all |
| 2. His/ her behavior toward another caregiver | yes | no | 1+ daily | once daily | 5-6x week | 3-4x week | 1-2x week | once a month | Extremely | very | somewhat | a little | not at all |
| 3. His/her behavior toward his/her (step) brothers and sisters | yes | no | 1+  daily | once daily | 5-6x week | 3-4x week | 1-2x week | once a month | Extremely | very | somewhat | a little | not at all |
| 4. His/her attitude/manners | yes | no | 1+  daily | once daily | 5-6x week | 3-4x week | 1-2x week | once a month | Extremely | very | somewhat | a little | not at all |
| 5. Him/her getting up in the mornings | yes | no | 1+ daily | once daily | 5-6x week | 3-4x week | 1-2x week | once a month | Extremely | very | somewhat | a little | not at all |
| 6. Him/her getting to school on time | yes | no | 1+ daily | once daily | 5-6x week | 3-4x week | 1-2x week | once a month | Extremely | very | somewhat | a little | not at all |
| 7. TV/internet/cell phone | yes | no | 1+ daily | once daily | 5-6x week | 3-4x week | 1-2x week | once a month | Extremely | very | somewhat | a little | not at all |
| 8. Him/her lying | yes | no | 1+ daily | once daily | 5-6x week | 3-4x week | 1-2x week | once a month | Extremely | very | somewhat | a little | not at all |
| 9. His/her chores (what they are, how they should be done, etc.) | yes | no | 1+ daily | once daily | 5-6x week | 3-4x week | 1-2x week | once a month | Extremely | very | somewhat | a little | not at all |
| 10. His/her health habits (eating, cleanliness, brushing teeth, etc.) | yes | no | 1+ daily | once daily | 5-6x week | 3-4x week | 1-2x week | once a month | Extremely | very | somewhat | a little | not at all |
| 11. His/her religious training | yes | no | 1+ daily | once daily | 5-6x week | 3-4x week | 1-2x week | once a month | Extremely | very | somewhat | a little | not at all |
| 12. His/her money (how much allowance, how money is spent) | yes | no | 1+ daily | once daily | 5-6x week | 3-4x week | 1-2x week | once a month | Extremely | very | somewhat | a little | not at all |
| 13. His/her cursing | yes | no | 1+ daily | once daily | 5-6x week | 3-4x week | 1-2x week | once a month | Extremely | very | somewhat | a little | not at all |

| In the past month have you and your child disagreed over any of these issues? (circle YES or NO) | | | IF YES, how often have you disagreed?  (circle one) | | | | | | IF YES, how **bad** are the disagreement(s)?  (circle one) | | | | |
| --- | --- | --- | --- | --- | --- | --- | --- | --- | --- | --- | --- | --- | --- |
| 14. His/her personal appearance (choice of clothes, haircuts, etc.) | yes | no | 1+ daily | once daily | 5-6x week | 3-4x week | 1-2x week | once a month | Extremely | very | somewhat | a little | not at all |
| 15. His/her household routines (bedtime, mealtimes, etc.) | yes | no | 1+ daily | once daily | 5-6x week | 3-4x week | 1-2x week | once a month | Extremely | very | somewhat | a little | not at all |
| 16. His/her homework (completing homework, quality of homework) | yes | no | 1+  daily | once daily | 5-6x week | 3-4x week | 1-2x week | once a month | Extremely | very | somewhat | a little | not at all |
| 17. His/her school grades | yes | no | 1+  daily | once daily | 5-6x week | 3-4x week | 1-2x week | once a month | Extremely | very | somewhat | a little | not at all |
| 18. His/her behavior in school | yes | no | 1+ daily | once daily | 5-6x week | 3-4x week | 1-2x week | once a month | Extremely | very | somewhat | a little | not at all |
| 19.Him/ her making too much noise at home | yes | no | 1+ daily | once daily | 5-6x week | 3-4x week | 1-2x week | once a month | Extremely | very | somewhat | a little | not at all |
| 20. Him/her playing the stereo, radio, TV too loudly | yes | no | 1+ daily | once daily | 5-6x week | 3-4x week | 1-2x week | once a month | Extremely | very | somewhat | a little | not at all |
| 21. How his/her free time is spent | yes | no | 1+ daily | once daily | 5-6x week | 3-4x week | 1-2x week | once a month | Extremely | very | somewhat | a little | not at all |
| 22. His/her choice of friends | yes | no | 1+ daily | once daily | 5-6x week | 3-4x week | 1-2x week | once a month | Extremely | very | somewhat | a little | not at all |
| 23. Keeping his/her room tidy | yes | no | 1+ daily | once daily | 5-6x week | 3-4x week | 1-2x week | once a month | Extremely | very | somewhat | a little | not at all |
| 24. Bothering you when you want to be left alone | yes | no | 1+ daily | once daily | 5-6x week | 3-4x week | 1-2x week | once a month | Extremely | very | somewhat | a little | not at all |
| 25. Something you may do that bothers your child and you argue about. | yes | no | 1+ daily | once daily | 5-6x week | 3-4x week | 1-2x week | once a month | Extremely | very | somewhat | a little | not at all |

**References**

Bentler, P. M. (1990). Comparative fit indexes in structural models. Psychological Bulletin, 107(2), 238–246. https://doi.org/10.1037/0033-2909.107.2.238

Browne, M. W., & Cudeck, R. (1993). Alternative ways of assessing model fit. In K. A. Bollen & J. S. Long, Testing Structural Equation Models (pp. 136–162). Sage Publications.

Cowan, P., & Cowan, C. (1992). Parenting style ratings: School children and their families project. Berkeley: University of California.

Lindahl, K. M., & Malik, N. M. (2000). The system for coding interactions and family functioning. Psychology Press.

Thambipillai, P., Vine, V., Vanwoerden, S., Byrd, A. L., Jennings, J. R., & Stepp, S. D. (under review). Embodied parenting cognition: Parenting sense of competence and dynamic physiological self-regulation before and during conflict with adolescents.
